# Development of a construct-based risk assessment framework for genetic engineered crops

**DOI:** 10.1007/s11248-016-9955-3

**Published:** 2016-06-23

**Authors:** M. P. Beker, P. Boari, M. Burachik, V. Cuadrado, M. Junco, S. Lede, M. A. Lema, D. Lewi, A. Maggi, I. Meoniz, G. Noé, C. Roca, C. Robredo, C. Rubinstein, C. Vicien, A. Whelan

**Affiliations:** 1 Bayer SA, Ricardo Gutierrez 3652, CP 1605, Munro, Buenos Aires Argentina; 2 Biotechnology Directorate, Secretariat of Value Adding, Av. Paseo Colón 922, 2nd, Of. 247, CP 1063, Ciudad Autonoma de Buenos Aires, Argentina; 3 Indear, Ocampo 210 bis Predio CCT Rosario (2000), Rosario, Santa Fe Argentina; 4 Monsanto Argentina, Maipu 1210, CP 1006, Ciudad Autonoma de Buenos Aires, Argentina; 5 National Agri Food Health and Quality Service, SENASA, Azopardo 1020, 1st, CP 1107, Ciudad Autonoma de Buenos Aires, Argentina; 6 BASF Argentina, Tucuman 1, 18th, CP 1049, Ciudad Autonoma de Buenos Aires, Argentina; 7 National Scientific and Technical Research Council, CONICET, Av. Rivadavia 1917, C1033AAJ Ciudad Autonoma de Buenos Aires, Argentina; 8 National University of Quilmes, Roque Sáenz Peña 352, CP 1876, Bernal, Buenos Aires Argentina; 9 National Agricultural Research Institute, INTA, Nicolas Repetto y de los Reseros s/n, CP 1686, Hurlingham, Buenos Aires Argentina; 10 Syngenta Agro, Av. Libertador 1855, CP 1638, Vicente Lopez, Buenos Aires Argentina; 11 Dow Agroscience SA, Cecilia Grierson 355, CP 1107, Ciudad Autonoma de Buenos Aires, Argentina; 12 Chacra Experimental Agricola Santa Rosa, Camino Vecinal Nº 8, Km 6, CP 4531, Colonia Santa Rosa, Salta Argentina; 13 ILSI Argentina, Ave Santa Fe 1145, 4th, C1059ABF Ciudad Autonoma de Buenos Aires, Argentina; 14 University of Buenos Aires and CERA, Sr Consultant, Av. San Martín 4453, CP 1417, Ciudad Autonoma de Buenos Aires, Argentina

**Keywords:** Genetic engineering, Risk assessment, Construct similarity, Familiarity, Problem Formulation

## Abstract

Experience gained in the risk assessment (RA) of genetically engineered (GE) crops since their first experimental introductions in the early nineties, has increased the level of familiarity with these breeding methodologies and has motivated several agencies and expert groups worldwide to revisit the scientific criteria underlying the RA process. Along these lines, the need to engage in a scientific discussion for the case of GE crops transformed with similar constructs was recently identified in Argentina. In response to this need, the Argentine branch of the International Life Sciences Institute (ILSI Argentina) convened a tripartite working group to discuss a science-based evaluation approach for transformation events developed with genetic constructs which are identical or similar to those used in previously evaluated or approved GE crops. This discussion considered new transformation events within the same or different species and covered both environmental and food safety aspects. A construct similarity concept was defined, considering the biological function of the introduced genes. Factors like environmental and dietary exposure, familiarity with both the crop and the trait as well as the crop biology, were identified as key to inform a construct-based RA process.

## Introduction

Risk assessment (RA) of genetically engineered crops has been underway for almost 25 years in many parts of the world (James 2014). Argentina was one of the first countries to implement a regulatory oversight process for GE crops (Burachik 2010) through the creation of the National Biosafety Advisory Commission for Agricultural Biotechnology (“CONABIA”), back in 1991.^1^ The increased level of familiarity with these methodologies and products, gained through new scientific knowledge, experimental evidence and cumulative experience, has motivated several agencies worldwide—including Argentina´s Biotechnology Directorate—to hold periodical discussions to update and refine regulatory criteria (Ministerio de Agricultura and Ganadería y Pesca 2013a, b; Yankelevich 2012).

A key issue that has emerged, is the extent to which new events transformed with the same or similar constructs need to be assessed for safety. Along this line, a guideline for a simplified assessment of “identical or essentially similar constructs” has recently been issued in Argentina (Ministerio de Agricultura and Ganadería y Pesca 2013b). Similar approaches exist in other countries with varied scopes and degrees of application (see Table 1), In order to address the scientific discussion around science based RA criteria for the simplified treatment of these cases, the International Life Sciences Institute (ILSI Argentina) convened a tripartite Working Group (WG) integrated by scientists from academia, government and industry, to discuss a science-based evaluation approach for the assessment of GE crops developed with genetic constructs identical or similar to those used in previously evaluated or approved GE crops.

**Table 1:** International precedents

Country (agency)	Scope of the simplified analysis	Reference
World Health Organization (WHO)	Gen/crop combinations which have been demonstrated to be substantially equivalent, can be used as reference for various crops and gene products. Gene products shown to be safe can be used in other crops without further testing, so long as increased exposure is not a safety concern	Report of a WHO Workshop World Health Organization (1995) http://apps.who.int/iris/bitstream/10665/58909/1/WHO_FNU_FOS_95.1.pdf
USA (EPA)	A DNA construct that has previously satisfied registration requirements in one crop may be part of the application submitted for use in another crop plant	Federal Insecticide, Fungicide, and Rodenticide Act (FIFRA) (2000)
USA (USDA-APHIS)	Extensions of non-regulated status based on a similarity of the new plant to an antecedent organism previously approved. Cases that could be considered similar are described.	Guidance on petitions for extensions of non-regulated status, 2015 http://www.aphis.usda.gov/wps/portal/aphis/ourfocus/biotechnology/sa_permits_notifications_and_petitions/sa_guidance_documents/ct_extensions/!ut/p/a0/04_Sj9CPykssy0xPLMnMz0vMAfGjzOK9_D2MDJ0MjDzd3V2dDDz93HwCzL29jAyMTPULsh0VAU1Vels!/
Brazil (CTNBio)	A new GMO of the same species with the same genetic construct used in a GMO with a previously granted favorable technical opinion	National Technical Biosafety Committee, (CTNBIO), Normative # 5 (March 12, 2008. Article 3). http://www.ctnbio.gov.br/index.php/content/view/12857.html
Canada (Canadian Food Inspection Agency)	“Re-transformation” with identical construct(s) as a previously authorized plant of the same species which conveys the same novel trait	Canadian Food Inspection Agency, (CFIA) 2008 Directive 98-08 http://www.inspection.gc.ca/plants/plants-with-novel-traits/applicants/directive-94-08/eng/1304475469806/1304475550733http://www.inspection.gc.ca/plants/plants-with-novel-traits/applicants/directive-94-08/eng/1304475469806/1304475550733
Argentina (Biotechnology Directorate)	Special treatment, on a case-by-case basis, of crops transformed with constructs that are identical or essentially similar to other constructs present in crops which have passed a risk assessment review or are already commercially approved. No new experimental field trials would be required by default	Minagri, SAGyP, Resolución Nº 318/2013 http://www.minagri.gob.ar/site/agregado_de_valor/biotecnologia/60-SOLICITUDES/___experimental/318-2013.pdf

The robustness of current RA systems, plus a growing body of evidence showing that domestication, conventional breeding (Osborn et al. 2007; Doebley et al. 2006; Lenser and Theißen 2013; Sang 2009; Koenig et al. 2013; Flint-Garcia 2013) and the intrinsic plasticity of plant genomes are greater sources of genetic changes than methodologies based on genetic engineering (Batista et al. 2008; Weber et al. 2012; Ricroch et al. 2011; Ricroch 2012; EFSA 2012; Kogel et al. 2010; Venkatesh et al. 2014), provided both context and technical background to this discussion.

The purpose of this work is to discuss the principles for the risk assessment of identical or similar constructs using accepted methodologies and available knowledge, and does not intend to present a detailed, prescriptive process, as these situations would be expected to be considerably diverse.

### International precedents

The design of a simplified approach for the safety assessment of similar or identical constructs has been discussed in several countries, where regulatory agencies and other organizations have issued comments, opinions or even specific guidelines, as summarized in Table 1.

In general, these guidelines specify the criteria to be met by a given case to be considered *similar* to a precedent one, and although most follow a case-by-case approach for eligibility, these criteria are applicable to a broad range of situations allowing a simplified, yet conclusive RA. As shown in Table 1, although the guideline issued in Argentina allows for a simplified treatment, it does not specifically define similarity, when referring to constructs that are “essentially similar”.

### Rationale

Three main aspects were considered in order to discuss science-based recommendations for a simplified RA of similar constructs: the potential unintended effects arising from the transformation process, the familiarity with the crop and the trait, and the specific RA considerations involved. On these grounds, a list of relevant questions was developed to guide a construct based, simplified RA.

These considerations assume that new events are transformed with constructs which are identical or similar (as defined below) to those already used in GE crops favorably reviewed or commercially approved by the competent authorities reviewing the new cases. Under this assumption, two situations were considered: new transformation events within the same species, or in different species. In both situations, the body of available knowledge and experience, in addition to targeted experimental evidence (a “basic set of data”), should allow for a simplified RA approach. For this, the Problem Formulation (PF) methodology (Wolt et al. 2010) aids in the review of the information already available from the previous RA and helps identify any new risk hypothesis that might need to be tested.

### Definitions

In order to frame the discussion, some definitions were adopted for coherence. To define *similar constructs,* it was agreed to consider biological functions rather than purely focus on sequence homology. Therefore, the following definitions were adopted:

*Event*: is the stable and simultaneous insertion into the plant genome, of one or more genes or DNA sequences as constitutive parts of a defined genetic construct.^2^
*Construct*: a set of nucleotide sequences designed to express certain phenotypic characteristics when introduced into the recipient organism.
*Similar constructs*: constructs designed to obtain the same phenotypic characteristic(s) in the recipient organism through the same biological mechanism(s). Different situations may fall under this definition that might trigger different data requirements. Accordingly, every similarity claim would have to be substantiated, as detailed in the following sections.

### Unintended effects of transformation

Genomic and physiological plasticity have been a driving force in the evolution of plant species, conferring them a high degree of adaptability (Schnell et al. 2014; Weber et al. 2012; Casacuberta et al. 2000; Ladics et al. 2014). Potential unintended effects of transgenesis have been attributed to several factors, such as the insertion interrupting genes or regulatory sequences, or to the somaclonal variation derived from tissue culture procedures. However, these sources of unintended changes are not different from those occurring in traditional breeding or through natural genome rearrangements (EFSA 2012; Venkatesh et al. 2014).

Insertional effects in GE plants have been recently reviewed by Schnell et al. (2015) who, based on the evidence accumulated on the nature and impact of genetic changes in plants, concluded that it is reasonable to use available knowledge and familiarity criteria to assist the pre-market assessments of GE plants and derived food and feed. This approach is also supported by evidence indicating that insertional effects associated with GE techniques are not different to the genetic changes that occur in conventionally bred plants and therefore should represent similar levels of risk (Weber et al. 2012; Van der Wiel et al. 2010; Kobayashi et al. 2004; EFSA 2012).

The generation of new open reading frames (ORFs) which may occur upon insertion of DNA sequences during transgenesis, can also occur in conventional breeding or result from natural genomic rearrangements. Although it is unlikely that these changes will result in the expression of novel proteins, most if not all regulatory authorities typically require that the molecular characterization of transgenic events include bioinformatic analyses to identify putative ORFs or potential expression products (Kovalic et al. 2012). Likewise, somaclonal variation, a well known phenomenon that can cause considerable genetic change in plant cells, is enhanced under tissue culture conditions and have been used as a source of genetic variation in conventional plant breeding (Cardone et al. 2008; Kaeppler et al. 2000).

Whichever the breeding process used, conventional or GE-assisted, unwanted phenotypes are routinely eliminated during selection for the desired traits (Schnell et al. 2014; Parrott et al. 2010) and therefore, focus should be put on the construct itself and on the expressed phenotype, when assessing identical or similar constructs. Regarding the transformation methods used to introduce similar constructs in different cases, this was not considered to be relevant to the biosafety assessment (Schnell et al. 2014; USDA-APHIS 2015).

### Familiarity

As applied to GE crops, familiarity has been described as “the knowledge gained through experience over time, that considers the nature of the crop that was modified, the characteristics of the trait that was introduced, the likely receiving environment for the GM crop, and the likely interactions between these” (OECD 1993).

The concept of familiarity was jointly developed by different groups (National Research Council 1989; Tiedje et al. 1989; OECD 1993) and is a key approach to identify hazards and evaluate risks as well as to inform management practices based on identified risks (Nickson and McKee 2002).

Experience in different geographies, scientific literature, and empirical data, all provide risk assessors with a solid context for assessing familiarity (Garcia-Alonso et al. 2014; Conner et al. 2003). In the case of constructs which are identical or similar to others used in previously assessed transformation events, familiarity is instrumental to inform and support the RA of new events. For example, familiarity can draw from the extensive experience gained with some traits, like herbicide-tolerant and insect-resistant events, which have been subjected to over 600 assessments, obtained dozens of commercial approvals worldwide (94 as of 2015) and have been widely adopted and consumed since 1996 at the global scale (James 2015; CERA 2015); Van Eenennaam and Young 2014; Koch et al. 2015).

### Problem formulation considerations

Problem formulation (PF) is the first step in a RA process, whereby policy goals, scope, study plans, assessment endpoints and measurement methodologies are condensed into an explicitly stated problem and its approach for analysis. Applied to GE crops, a rigorous PF producing an analysis plan describing relevant exposure scenarios and their potential outcomes, assures the relevance of the RA for decision-making (Wolt et al. 2010; EFSA 2010; Tepfer et al. 2013). Although generally applied to assess the environmental risk assessment (ERA) of transgenic crops, this methodology can be further extended to the food and feed safety aspects of GE crops assessment (Garcia Alonso 2013). Typically, through PF, the relevant questions/concerns to be addressed are defined; the available information and data that must be generated are identified, together with the best analysis plan and endpoints to be measured to respond to those concerns.

This exercise allows to define risk hypotheses that are relevant to the RA. A risk hypothesis describes the way in which a hazard is verified as a risk, addressing protection goals, which may be defined in laws, statutes, regulations, or guidance. Risk hypotheses must be biologically plausible and the hazard and the risk must be connected by discrete steps (the path to harm) that can be tested individually. In this way, the risk hypotheses are translated into one or more experimental hypotheses that can be used for testing and corroboration. (Wolt et al. 2010; Paes de Andrade et al. 2012).

### Risk assessment considerations

Risks to human or animal health and the environment would derive essentially from the particular characteristics of the crop/trait combination, the related exposure factors (extent of cultivation and consumption) and the specific agronomic practices/processing or intended uses that might influence these factors. These considerations should be part of the Problem Formulation exercise as the first step in the RA process.

The simplified approach for identical or similar constructs assumes, and builds on, the availability of prior environmental and food/feed safety assessments that should be used as the reference for subsequent evaluations. Accordingly, the risk assessment should then focus on two aspects that will help identify new or different risk hypotheses for the new GE crop: (a) Biology of the crop: a key aspect, particularly for different species transformed with similar constructs, as the new crop could lack a history of cultivation in the receiving environment or may have wild relatives that need to be considered in the ERA. On the food safety aspect, changes in hazards known to be intrinsically associated with a crop (*e.g.*, glycoalkaloids in potato, antinutrients in legumes or erucic acid in canola) should be assessed, irrespective of whether conventional or GE breeding has been used. The Consensus Documents Series on the biology of different crops (OECD, 2006) are a good source of information; (b) Environmental and dietary exposure: the extent of cultivation, particular agronomic practices (if different from events used as precedents), dietary intake, and intended uses should be considered.

### Relevant questions

A list of guiding questions was developed to assist in a construct based, simplified RA process. These questions should be applicable to new transformation events within the same or different species, using identical or similar genetic constructs and should help to: (a) decide if the case can be analyzed using a simplified approach, assuming that prior GE events with the same or similar constructs have been favorably reviewed or have been commercially approved and (b) identify the relevant questions/concerns that have to be addressed to formulate a plausible risk hypothesis for the new crop/trait combination, considering factors like crop biology, familiarity and exposure scenarios.
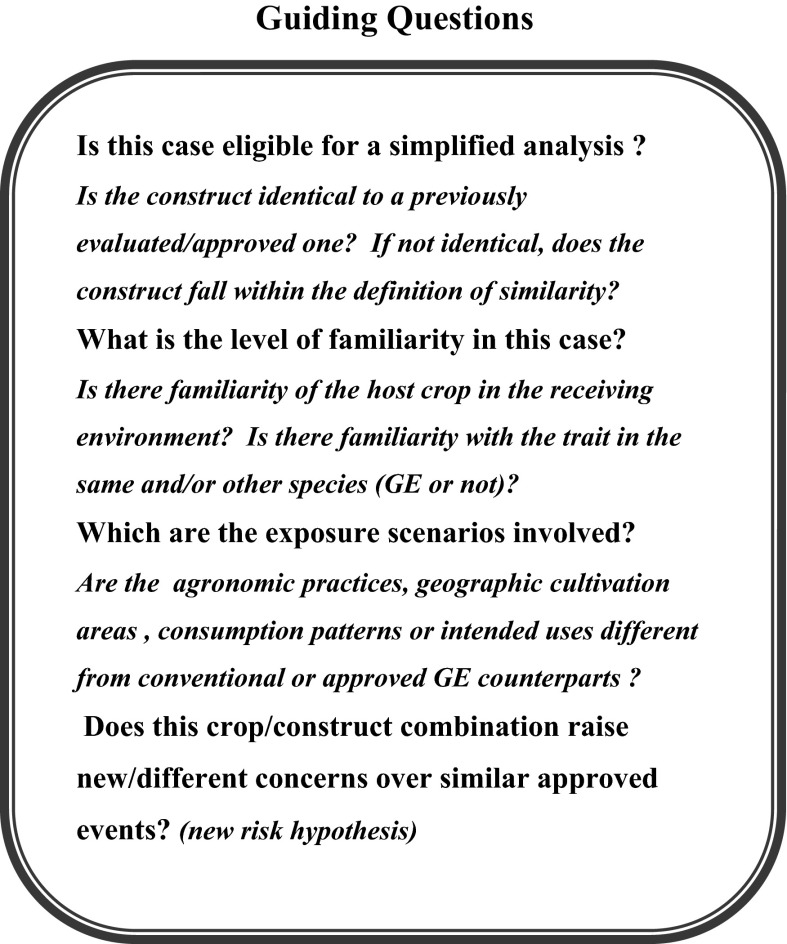


Under a simplified assessment context, applicants would have to provide a description that substantiates the similarity claim, including information on the genetic elements of the construct, expression products, conferred phenotype and a bioinformatic analysis of protein products (in case not identical to prior events). In addition, eligible cases for a simplified treatment will require a basic set of data that will be typically used to validate the use of prior assessments. This basic set of data, being confirmatory in nature, would conform to what is known as a “bridging” approach (see below). However, if the RA leads to the formulation of new risk hypotheses, additional data will have to be generated to test those hypotheses. When no new risk hypotheses are identified, no additional information would be required.

### Bridging experimental evidence

The bridging methodology for RA is a confirmatory approach that builds on available data to confirm the safety of new events, focusing on a few targeted, specific assessment endpoints. The following aspects were discussed as relevant to a bridging approach that would be applied on cases deemed eligible:

- *Molecular characterization* although molecular analysis is not predictive of potential unintended effects *per se*, it ensures that the developer has appropriately characterized the genetic modification and that the desired trait is attributable to the integrated construct. In addition, sequence analysis of the inserts and flanking regions, which allows for the specific identification of the event, it is also generally prescribed to identify putative ORFs that may have been created by the insertion. With regards to protein expression products, it will be appropriate to provide a bioinformatic analysis for proteins expressed from similar constructs that are not identical. Proteins that do perform the same function or catalyze the same biochemical reactions (and therefore conform to the definition) could be derived from different genes with various degrees of homology to the previously assessed proteins and therefore may trigger additional requirements, in particular if there is no history of safe use as food (Hammond et al. 2013; USDA-APHIS 2015).
- *Expression* protein expression level ranges can be informative in terms of exposure. This information could be relevant in some specific cases, depending on the crop and in terms of environmental or dietary exposure. In particular, in the ERA for traits with effects on target organisms, such as insect protection traits, effects on non-target organisms (NTO) can become significant under high expression contexts.
- *Composition* Depending on the transformation objective, compositional measurements would not be generally relevant for the assessment of new events within the same species. However, if the transformation goal is to alter composition, as in nutritional or other metabolic modifications, and if specific risk hypotheses are formulated that are contingent on compositional changes, compositional studies may have to be performed, together with the assessment of potential dietary impacts. The need to measure key nutrients, anti-nutrients or toxicants will depend on the relevance of the crop as a significant source for these. If available, the OECD Consensus Documents series on crop composition can be a reference to select the most relevant components to be measured (OECD 2002–2012). When sufficient information on the crop´s natural compositional variability ranges is available, data from the new events could be compared against these ranges (ILSI 2014.)
- *Phenotypic/agronomic data* A description of the selection process for the lead event, along with any additional selection during subsequent breeding, can add significantly to the weight of evidence in support of a construct based RA. In the case of different species with the same insect-protection trait, information about relevant NTO and/or beneficial species will be required on a crop/trait specific basis to assess the relevance of available lower tier studies to the new event (Romeis et al. 2008, 2011, 2013).

**Fig. 1:**
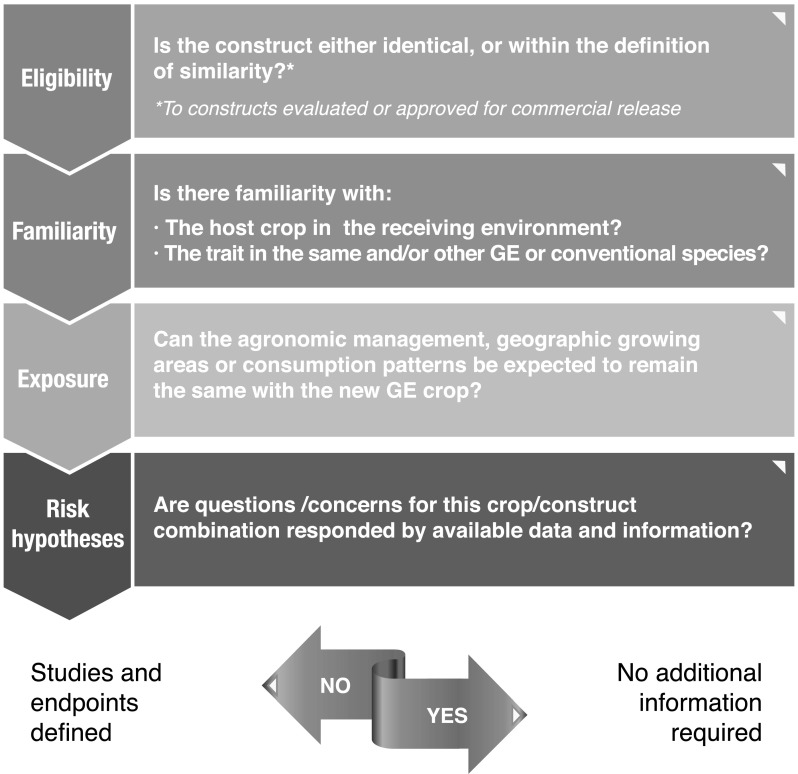
Risk assessment approach for identical or similar constructs. Affirmative answers to all questions indicate that a simplified RA is justified and no additional RA is required. Any negative answers may call for additional RA. All cases will need to provide a full description of the event and a basic set of data. The type and extent of data will be defined on a case by case basis (see text)

The need for information on some or all of the aspects defined above, as well as the scope and level of detail required, will depend on the case under study. A schematic description of the suggested construct based RA methodology is depicted in Fig. 1.

## Case examples

Two different cases were subjected to the RA exercise proposed here, to test the approach. For simplicity, Argentina´s agricultural production areas were taken as the receiving environments.

- *Case* 1 new transformation event(s) of the same crop species: herbicide-tolerant sugarcane

For vegetatively propagated crops like sugarcane, additional cultivars transformed with the same or similar constructs would be the most common situation eligible for a simplified approach. This is a high familiarity scenario, in which the RA would fundamentally rely on the evaluation done for the original, approved event. Considering the level of familiarity with the crop and the available information about the construct, focus will be put on the assessment of this crop/trait combination in the receiving environment. As long as the same GE trait in the new variety is not expected to alter the cultivation pattern or the intended uses of the sugarcane varieties and therefore, the exposure scenarios, then only the molecular characterization of the new events would be required to validate the extension of the original RA to this case (or cases), as the herbicide tolerance trait would not involve NTO considerations. As mentioned, the RA will also consider the selection process and the phenotypic behavior of the new varieties.

- *Case* 2 new transformation event(s) of a different crop species: insect-resistant soybean

This example considers a construct introduced in soybean, that is similar to a construct used in commercially approved GE-corn. The new construct falls within the definition of similarity: it contains a gene encoding an insecticidal protein which confers insect protection through the same biological mechanism (i.e. same binding target). It uses a coding sequence that is highly familiar and only has minor sequence changes to enhance expression efficiency in soybean; the regulatory sequences are identical to the ones used in the original construct and the selectable marker is different, but well known and widely used in other approved GE crops.

In this example, there is familiarity with the trait and the crop in the receiving environment; the trait is present in previously assessed GE corn and also in other corn events which are grown in the same environments as soybean. In turn, soybean is a widely cultivated crop in the country, and there are not wild relatives.

The focus of the RA process in this case, is on the biology of the host crop (soybean) and its history of cultivation in the receiving environment, as well as on environmental and dietary exposures. In this case, the basic set of data required to validate the use of the RA data from GE corn, will include: (1) molecular characterization of the inserted DNA and bioinformatic analysis of the protein product, (2) a description of the selection process used to obtain the new soybean event (in soybean, backcross introgression assisted by molecular markers is commonly performed), (3) expression level ranges in relevant tissues and (4) data on anti-nutrient levels, compared against available soybean composition databases.

Nevertheless, as this is an insect resistance trait, it may need additional evidence to complete the RA. For example, if certain non-target and/or beneficial organisms are known to interact specifically with soybean and not with corn, it could be hypothesized that, when exposed to the GE soybean, these organisms could be affected by the action of the newly expressed protein. Therefore, additional data would be required (typically, Tier 1 study) to address this concern (Romeis et al. 2008, 2011, 2013). See Table 2 for a summary of these cases.

**Table 2:** RA summary for two construct based evaluation situations (*HT* herbicide tolerant, *IR* insect resistant, *R* required, *NR* not required)

Guiding questions	Case 1 HT sugarcane	Case 2 IR soybean
Is the construct identical or similar to a previously evaluated/approved one?	Yes	Yes
Is the host crop familiar to the receiving environment?	Yes	Yes
Is there experience with the trait in the same and/or other species (GE or not)?	Yes	Yes
Is it expected that management, geographic growing areas or consumption patterns remain the same?	Yes	Yes
Are intended uses similar to those from available cultivars?	Yes	Yes
Are the questions raised by this crop/construct combination responded by available data?	Yes	No
Basic set of data		
Molecular characterization	R	R
Expression levels range	NR	R
Composition (key nutrients/anti-nutrients –crop specific)	NR	R
Selection process information	R	R
Additional evidence to complete the RA	NR	R (Tier 1 data)

## Conclusions

The framework for a simplified approach draws on considerations on the likelihood of unintended effects of the GE methods and on familiarity, and allows to determine eligibility of a case, along with the information requirements that would allow to make science-based decisions about the safety of the new cases. A construct similarity concept was defined based on functional similarity, that can be used for a simplified RA of GE crops.

If new risk hypotheses are generated as a result of this exercise, a specific set of additional data would be required, on a case by case basis.

In addition to major crops, including those that need to be transformed *de novo* to develop new varieties (i.e. vegetatively propagated crops), this framework can aid to perform RA for specialty or ornamental crops, tree species, etc., including the so called “orphan crops”, of regional or local interest (Falck Zepeda and Cohen 2006). In this sense, initiatives like inter-agency collaborations, joint reviews or mutual recognition of RA reviews could greatly facilitate the use of this approach, in particular in developing countries (Bartholomaeus et al. 2015).

It is expected that the construct based approach here presented can adequately be applied building on previous knowledge and familiarity, without compromising the robustness of the RA, while minimizing or avoiding the review of redundant information and the use of limited resources.

## Footnotes

1 www.minagri.gob.ar/site/agregado_de_valor/biotecnologia/20-CONABIA/index.php.

2 As defined in: http://www.minagri.gob.ar/site/agregado_de_valor/biotecnologia/60-SOLICITUDES/___experimental/_archivos/resolucion%20OVGM%20701-2011.pdf.
